# 454 Pyrosequencing-based assessment of bacterial diversity and community structure in termite guts, mounds and surrounding soils

**DOI:** 10.1186/s40064-015-1262-6

**Published:** 2015-09-02

**Authors:** Huxley M. Makonde, Romano Mwirichia, Zipporah Osiemo, Hamadi I. Boga, Hans-Peter Klenk

**Affiliations:** Leibniz-Institute DSMZ-German Collection of Microorganisms and Cell Cultures, Inhoffenstraße 7B, 38124 Brunswick, Germany; Pure and Applied Sciences, Technical University of Mombasa, P.O.Box 90420-80100, Mombasa, Kenya; Department of Biological Sciences, Embu University College, P.O.Box 6-60100, Embu, Kenya; Zoology, Jomo Kenyatta University of Agriculture and Technology, P.O.Box 62000-00200, Nairobi, Kenya; Botany, Jomo Kenyatta University of Agriculture and Technology, P.O.Box 62000-00200, Nairobi, Kenya; School of Biology, Newcastle University, Ridley Building, Newcastle upon Tyne, NE1 7RU UK

**Keywords:** Termites gut symbionts, 454 Pyrosequencing, OTUs, Macrotermitinae

## Abstract

Termites constitute part of diverse and economically important termite fauna in Africa, but information on gut microbiota and their associated soil microbiome is still inadequate. In this study, we assessed and compared the bacterial diversity and community structure between termites’ gut, their mounds and surrounding soil using the 454 pyrosequencing-based analysis of 16S rRNA gene sequences. A wood-feeder termite (*Microcerotermes* sp.), three fungus-cultivating termites (*Macrotermes michaelseni*, *Odontotermes* sp. and *Microtermes* sp.), their associated mounds and corresponding savannah soil samples were analyzed. The pH of the gut homogenates and soil physico-chemical properties were determined. The results indicated significant difference in bacterial community composition and structure between the gut and corresponding soil samples. Soil samples (Chao1 index ranged from 1359 to 2619) had higher species richness than gut samples (Chao1 index ranged from 461 to 1527). The bacterial composition and community structure in the gut of *Macrotermes michaelseni* and *Odontotermes* sp. were almost identical but different from that of *Microtermes* and *Microcerotermes* species, which had unique community structures. The most predominant bacterial phyla in the gut were *Bacteroidetes* (40–58 %), *Spirochaetes* (10–70 %), *Firmicutes* (17–27 %) and *Fibrobacteres* (13 %) while in the soil samples were *Acidobacteria* (28–45 %), *Actinobacteria* (20–40 %) and *Proteobacteria* (18–24 %). Some termite gut-specific bacterial lineages belonging to the genera *Dysgonomonas*, *Parabacteroides*, *Paludibacter*, *Tannerella*, *Alistipes*, *BCf9*-*17 termite group* and *Termite Treponema cluster* were observed. The results not only demonstrated a high level of bacterial diversity in the gut and surrounding soil environments, but also presence of distinct bacterial communities that are yet to be cultivated. Therefore, combined efforts using both culture and culture-independent methods are suggested to comprehensively characterize the bacterial species and their specific roles in these environments.

**Electronic supplementary material:**

The online version of this article (doi:10.1186/s40064-015-1262-6) contains supplementary material, which is available to authorized users.

## Background

Termites (Isoptera) are a large and diverse group of soil macrofauna comprising of >2600 species worldwide (Ahmed et al. 2011). The greatest termite diversity is in Africa (Eggleton 2000), where they play diverse roles in semi-arid and humid ecosystems: As soil engineers, termites have an impact on the soil structure (Holt and Lepage 2000), which modifies the soil environment thereby controlling diversity and activity of other soil organisms (Jones et al. 1997). Their influence on the soil microbial component is due to their major construction activities of complex galleries and mounds, which partly contribute to soil heterogeneity in the tropical regions (Holt and Lepage 2000). The termite mound is made from a mineral matrix mixed with feces or saliva, depending on the termite species and forms a specific habitat for soil microbes since the physical and chemical properties are different from the surrounding soil (Brauman 2000; Holt and Lepage 2000).

The type of a mound constructed depends on the feeding habit of the termite species (Holt and Lepage 2000): Soil-feeders (subfamily *Termitinae*) build their mounds with fecal matter mixed with coarse, inorganic particles (Noirot and Darlington 2000) and have a limited effect on the surrounding soil of about 20 cm in depth and within a range of a few meters (Harry et al. 2001). However, the fungus-growing termites (subfamily *Macrotermitinae*) build their mounds using soil and clay cemented by salivary secretions, which make the mounds enriched with clay particles but impoverished in carbon (Harry et al. 2001). The nest-walls consist of organo-mineral aggregates, characterized by a low stability and thus mineralize easily (Garnier-Sillam et al. 1988). They have a wider range of activity on the surrounding soil of 1–3 m in depth and within a range of a 2–8 m (Harry et al. 2001), which may influence the soil properties and fertility. The question is whether the fungus-feeding termites induce soil microbial changes as those observed in soil-feeders (Harry et al. 2001; Fall et al. 2004; Roose-Amsaleg et al. 2004; Fall et al. 2007). This forms the principle objective of this study with a focus on bacterial community structure in the different environments (termite gut, associated mound and surrounding soil ecosystems).

Previously, studies on microbial communities between termite guts and mounds (Roose-Amsaleg et al. 2004; Fall et al. 2007), mounds (Fall et al. 2004) and termite mounds and surrounding soils (Holt 1996; Harry et al. 2001) indicated differences in the microbial community abundance. Moreover, the gut bacterial communities have been assessed by using traditional molecular methods such as Sanger sequencing-based analysis of 16S rRNA gene libraries or fingerprinting techniques (Schmitt-Wagner et al. 2003; Shinzato et al. 2005, 2007; Fisher et al. 2007; Fall et al. 2007; Mackenzie et al. 2007; Mathew et al. 2012; Makonde et al. 2013a). These results not only indicated high bacterial diversity in the guts, but also termite-specific bacterial lineages (Shinzato et al. 2005). Such methods, however, were often limited to the analysis of a relative small number of clones.

To compressively describe and compare the microbial community structure in different ecosystems, high-throughput methods (Droege and Hill 2008; Glenn 2011) are necessary. Recently, high resolution analyses from five genera of the *Macrotermitinae* revealed that community composition almost resembles host phylogeny and their gut microbiotas are distinct from those of other termites (Otani et al. 2014). Elsewhere, analysis of the gut environment and bacterial microbiota (Köhler et al. 2012) revealed functional compartmentation on wood-feeding higher termites (*Nasutitermes* spp.). In this study, we used 454 pyrosequencing-based analysis of the 16S rRNA gene region to assess and compare the bacterial diversity and community structure in the gut of termites, associated termite mounds and surrounding soil environments. This is the first study that attempts to comparatively assess the bacterial diversity and structure in termite gut and surrounding habitats using the high-throughput sequencing approach. The results indicated variation in bacterial diversity and structure in the different environments.

## Results

### Description of the samples

The pH of the gut homogenates was within the neutral range (pH 7–8). The soils were slightly acidic (pH range 5–7) with overall high sand (76 %) and a relative increase in clay content (30 and 20 %) in the two mounds compared to the corresponding savannah soil (27.5 and 2.5 %). Similarly, organic carbon (OC) and nitrogen (N) contents had overall slightly higher values in savannah soil (3.0 and 0.3 mg/g, respectively) compared to the mounds (2.0 and 0.2 mg/g, respectively). The C/N ratios ranged from 9 to 11 (see Additional file 1a).

### Distribution of phyla across the samples

**Fig. 1:**
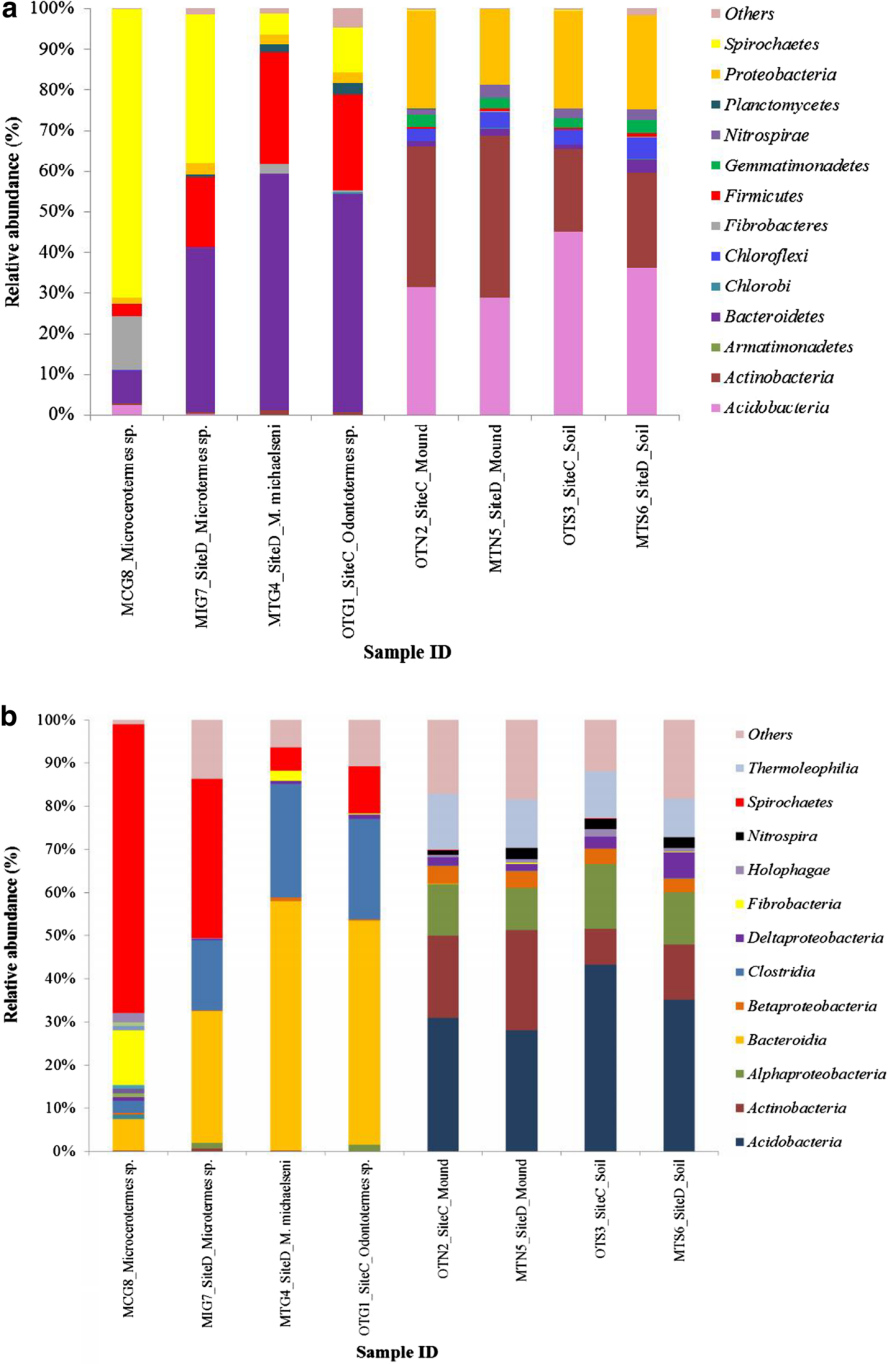
**a** Relative abundances of phylogenetic groups in the samples. **b** Relative abundances of bacterial groups (at class level) in the samples. *MCG8* *Microcerotermes* sp. gut homogenate, *MIG7* *Microtermes* sp. gut homogenate, *OTG1* *Odontotermes* sp. gut homogenate, *MTG4* *M. michaelseni* gut homogenate, *OTN2* soil from mound C of *Odontotermes* sp., *MTN5* soil from mound D of *M.* *michaelseni*, *MTS6* soil collected 3 m away from mound D, *OTS3* soil collected 3 m away from mound C. Phylogenetic groups accounting for ≤0.4 % of the analyzed sequences were included in the artificial group ‘others’

A total of 17, 528 reads were obtained for the bacterial samples. After quality filtering and chimera check 14, 301, the resulting sequences (≥300 bp) were clustered into 4, 157 operational taxonomic units [OTUs] (Table 1) at 3 % genetic distance according to the approach described by Huse et al. (2010). Taxonomic assignment of the resulting sequences against the SILVA database showed that a total of 21 phyla were represented and the major ones were: *Bacteroidetes*, *Acidobacteria*, *Spirochaetes*, *Actinobacteria*, *Proteobacteria*, *Firmicutes*, *Fibrobacteres* and *Chloroflexi* (Fig. 1a; Table 2). The other 13 phyla were represented at varying levels in one or more samples at <5 % of the effective sequences (Table 2).

**Table 1:** Number of sequences, observed OTUs, the estimated richness and diversity indices at 3 % dissimilarity threshold

Sample ID	Sample description	Reads before	Reads after	OTUs	Phyla	Classes	Richness and diversity indices				
							Chao1 index	ACE	Simpson (1/D)	Shannon	Fisher_alpha
OTG1	Site C *Odontotermes* sp. gut contents	2064	1677	552	11	20	1527.1	1049.9	1.0	5.2	151.0
OTN2	Site C *Odontotermes* sp. mound	1964	1609	593	13	41	2619.9	1737.6	1.0	5.3	190.1
OTS3	Site C *Odontotermes* sp. soil	1926	1598	591	14	35	2528.5	1980.7	1.0	5.3	203.0
MTG4	Site D *M. michaelseni* gut contents	2112	1652	423	11	23	818.0	637.9	1.0	5.0	106.8
MTN5	Site D *M.* *michaelseni* mound	2690	2216	645	12	44	1906.3	1331.5	1.0	5.3	187.7
MTS6	Site D *M.* *michaelseni* soil	2550	2194	605	16	51	1359.7	941.8	1.0	5.4	164.4
MIG7	Site D *Microtermes* sp. gut contents	2287	1863	487	12	23	1152.3	832.1	1.0	5.0	120.1
MCG8	*Microcerotermes* sp. gut contents	1935	1492	261	11	20	461.3	375.5	0.9	4.0	59.6
		17,528	14,301								

*MCG8* *Microcerotermes* sp. gut homogenate, *MIG7* *Microtermes* sp. gut homogenate, *OTG1* *Odontotermes* sp. gut homogenate, *MTG4* *M. michaelseni* gut homogenate, *OTN2* soil from mound C of *Odontotermes* sp., *MTN5* soil from mound D of *M.* *michaelseni*, *MTS6* soil collected 3 m away from mound D, *OTS3* soil collected 3 m away from mound C

**Table 2:** Relative abundances of phylogenetic groups (at phylum level) in the samples

Phylum	Termite gut				Mound		Savannah soil	
	MCG8	MIG7	MTG4	OTG1	OTN2	MTN5	OTS3	MTS6
*Acidobacteria*	2.3	0.2	0	0	*31.5*	*28.9*	*45.2*	*36.2*
*Actinobacteria*	0.6	0.6	1.1	0.6	*34.5*	*39.9*	*20.3*	*23.4*
Armatimonadetes	0	0	0	0	0	0	0	0.1
*Bacteroidetes*	*7.9*	*40.1*	*58.3*	*53.8*	1.5	1.6	1.1	3.2
Candidate division TM7	0	0	0	0	0.1	0	0	0
Candidate division WS3	0	0	0	0	0	0.1	0.2	0.3
Chlorobi	0.1	0.1	0.1	0.5	0	0.2	0.1	0.3
Chloroflexi	0.1	0.2	0	0	3	4	3.5	5.2
Cyanobacteria	0	0	0.5	0	0.4	0.1	0.1	0.2
Deferribacteres	0.1	0.1	0.3	1.4	0	0	0	0
Elusimicrobia	0	0.3	0	0.1	0.1	0	0	1
*Fibrobacteres*	*13.4*	0	2.4	0.3	0	0.2	0	0.1
*Firmicutes*	*3.1*	*17.5*	*27.5*	*23.7*	0.3	0.6	0.5	0.8
Gemmatimonadetes	0	0	0	0	3.2	2.7	2.4	3.5
Nitrospirae	0	0	0	0	1.3	3.1	2.3	2.5
Planctomycetes	0	0.6	2	2.9	0.2	0	0.1	0
*Proteobacteria*	1.4	2.8	2.2	2.6	*24*	*18.6*	*23.9*	*23.1*
SM2F11	0	0	0	0	0	0	0	0.1
*Spirochaetes*	*70.9*	*36.5*	*5.2*	*10.9*	0.1	0	0.2	0
Synergistetes	0.3	1.2	0.4	3.2	0	0	0	0
WCHB1-60	0	0	0	0	0	0	0.1	0.2

The most abundant phyla (≥5 % of the analyzed sequences) are shown in italics

*MCG8* *Microcerotermes* sp. gut homogenate, *MIG7* *Microtermes* sp. gut homogenate, *OTG1* *Odontotermes* sp. gut homogenate, *MTG4* *M. michaelseni* gut homogenate, *OTN2* soil from mound C of *Odontotermes* sp., *MTN5* soil from mound D of *M.* *michaelseni*, *MTS6* soil collected 3 m away from mound D, *OTS3* soil collected 3 m away from mound C

### Bacterial community structure across samples

Bacterial composition at the phylum level differed between the termite guts, mounds, and soil environments (Fig. 1a). Each environment was dominated by a particular phylum/phyla (≥5 % of the effective sequences). In the termite guts (MTG4, OTG1 and MIG7 samples), *Bacteroidetes* (>40 %) was the most abundant phylum while sample MCG8 was dominated by the phylum *Spirochaetes* (>70 %). Within the mounds (samples OTN2 and MTN5), the most abundant phyla were *Actinobacteria* (34–40 %), followed by *Acidobacteria* (28–32 %), whereas the corresponding soils (samples OTS3 and MTS6) were predominated by *Acidobacteria* (36–45 %). Four major phyla (*Bacteroidetes*, *Proteobacteria*, *Firmicutes* and *Actinobacteria*) were shared by all samples, but in different relative abundances (Fig. 1a; Table 2). Two phyla (*Deferribacteres* and *Synergistetes*) were exclusively detected in the gut samples while *Gemmatimonadetes*, *Nitrospirae*, *Armatimonadetes*, *Candidate division TM7*, *Candidate division WS3*, *SM2F11* and *WCHB1*-*60* were only detected in the mounds and surrounding soil in one or more samples (Fig. 1a; Table 2). Moreover, differences in bacterial community members were observed at the class level (Fig. 1b). At the family level, members from different families *Porphyromonadaceae*, *Rikenellaceae*, *Ruminococcaceae* and *Lachnospiraceae* were more abundant within the guts than in the mounds and savannah soil samples. However, the wood-feeding termite (sample MCG8) was dominated by the family *Spirochaetaceae* and *termite gut group*, representing 70 and 12 % of the effective sequences, respectively (Additional file 2).

**Fig. 2:**
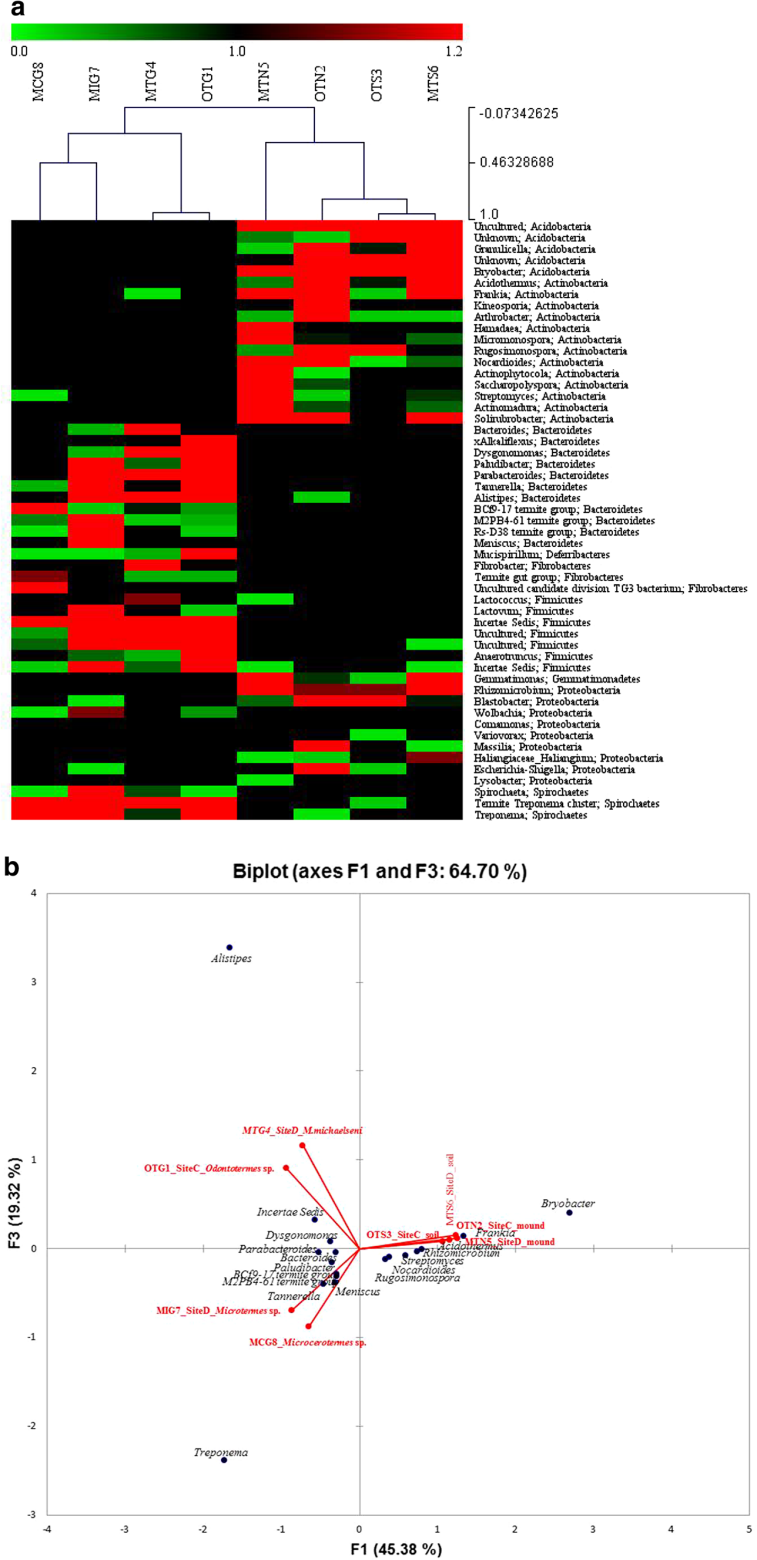
**a** Heatmap shows hierarchical clustering of taxa (relative abundance ≥1.0 % of the analyzed sequences). The *scale bar* represents color saturation gradient based on the relative abundances of the bacterial genera. The dendrogram at the *top* shows the weighted Euclidean distance analysis of community similarity. Classification is presented at the genus and phylum levels. **b** PCA of bacterial communities based on the relative abundances (≥2.3 %) of selected genera. The *vectors* indicate the direction and impact of each genus on the overall variance. R squared (r^2^) = 0.49. *MCG8* *Microcerotermes* sp. gut homogenate, *MIG7* *Microtermes* sp. gut homogenate, *OTG1* *Odontotermes* sp. gut homogenate, *MTG4 M. michaelseni* gut homogenate, *OTN2* soil from mound C of *Odontotermes* sp., *MTN5* soil from mound D of *M.* *michaelseni*, *MTS6* soil collected 3 m away from mound D, *OTS3* soil collected 3 m away from mound C

**Fig. 3:**
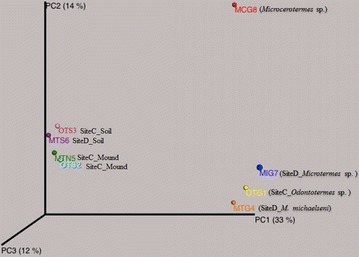
A 3 dimensional PCoA plot showing the degree of similarity of bacterial communities on termite guts, mounds and soil samples. R squared (r^2^) = 0.69. *MCG8 Microcerotermes* sp. gut homogenate, *MIG7* *Microtermes* sp. gut homogenate, *OTG1* *Odontotermes* sp. gut homogenate, *MTG4* *M. michaelseni* gut homogenate, *OTN2* soil from mound C of *Odontotermes* sp., *MTN5* soil from mound D of *M.* *michaelseni*, *MTS6* soil collected 3 m away from mound D, *OTS3* soil collected 3 m away from mound C

At high taxonomic resolution, there were salient differences in relative abundance of majority genera across the samples. Within the gut samples, there were 13 genera with known members that were represented with a value ≥2 % in one or more samples (Additional file 2). Notably, the genus *Termite Treponema cluster* was the most abundant in sample MCG8 (>50 %), while the genus *Treponema* (≥19 %) was more abundant in MCG8 and MIG7 than in samples OTG1 and MTG4, which were dominated by the genus *Alistipes* (>30 %) (Additional file 2). For the mounds and savannah soil samples, there were nine genera with known members that were represented by a value ≥2 % in one or more samples. They included; *Bryobacter*, *Acidothermus*, *Frankia*, *Hamadaea*, *Rugosimonospora*, *Nocardioides*, *Streptomyces*, *Rhizomicrobium* and *Blastobacter* (Additional file 2). Clustering of samples based on community similarity clustered the gut and soil samples separately (Figs. 2a, 3). The gut samples (MTG4 and OTG1) had identical communities, thus, clustered together compared to MCG8 and MIG7 samples. Likewise, the mound samples (MTS6 and OTS3) had more similar communities compared to surrounding soil samples (OTS3 and OTN2).

### Bacterial diversity and richness

**Fig. 4:**
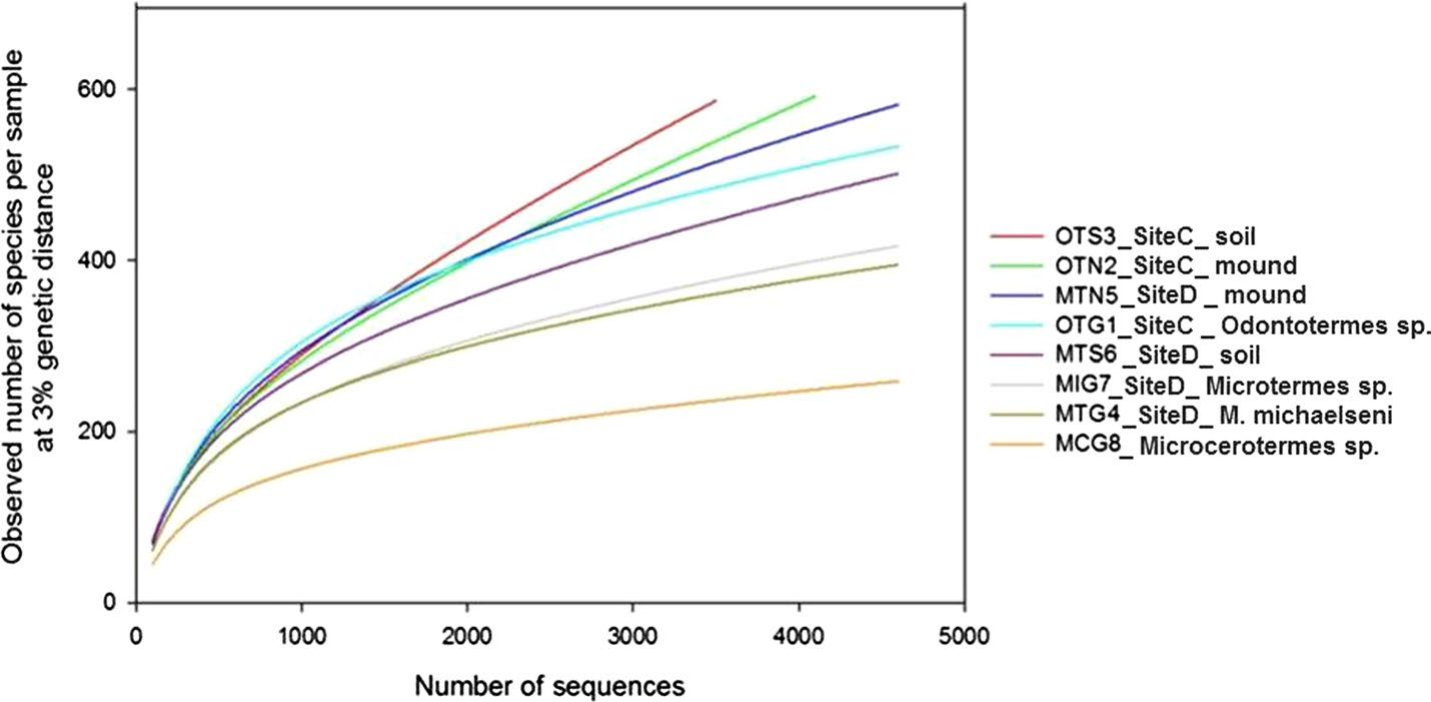
Rarefaction curves indicating the observed number of operational taxonomic units (OTUs). The samples are marked by *different colors*. *MCG8* *Microcerotermes* sp. gut homogenate, *MIG7 Microtermes* sp. gut homogenate, *OTG1* *Odontotermes* sp. gut homogenate, *MTG4* *M. michaelseni* gut homogenate, *OTN2* soil from mound C of *Odontotermes* sp., *MTN5* soil from mound D of *M.* *michaelseni*, *MTS6* soil collected 3 m away from mound D, *OTS3* soil collected 3 m away from mound C

Bacterial diversity and richness for the selected sequences from each sample (Table 1) was evaluated by rarefaction as shown in Fig. 4 and Additional file 1b. At 3, 5 and 10 % sequence divergence, most rarefaction curves did not reach saturation, indicating that the surveying efforts did not fully cover the extent of taxonomic diversity at these genetic distances, but a substantial fraction of the bacterial diversity within individual samples was assessed. The diversity measures indicated that MTN5 had the most genus-level taxa (645, Table 1) and MCG8 the least (261, Table 1), that OTN2 was richest (Chao 1 index), while MCG8 was poorest. Despite variation in community composition, Simpson (1/D) and Shannon indices were similar across communities, ranging from 0.9 to 1.0 and 4.0 to 5.4, respectively. Comparison between any pair of bacterial communities using unweighted UniFrac PCoA (Fig. 3) showed a distinct clustering by environment but the *p* value of 0.283 and R value of 0.091 indicated that the grouping of samples is weak. For instance, samples OTG1 and MTG4 clustered together (Fig. 3), indicating similarities in the two guts. Sample MIG7 was slightly away from OTG1 and MTG4 meaning that its bacterial communities and community structure are different from the two. Sample MCG8 was very distinct and far away from the other gut samples, implying that its bacterial communities are unique to those of fungus-cultivating termites. Likewise, the mound samples (OTN2 and MTN5) and soils samples (OTS3 and MTS6) clustered together (Figs. 2a, 3), indicating that the bacterial community structure of the sample types was almost identical. The PCA (Fig. 2b), indicated that the relative abundances of *Alistipes*, *Treponema*, *Bryobacter* and *Frankia* are the major effect determining the overall variance of the genus compositions in the samples. Differences regarding the other genera detected in the samples are minimal. *Alistipes* abundance increases in the direction of the *M. michaelseni* (sample MTG4) and *Odontotermes* sp. (sample OTG1), while abundance of *Treponema* increases towards *Microcerotermes* sp. (sample MCG8) and *Microtermes* sp. (sample MIG7). *Bryobacter* and *Frankia,* however, increases towards the mounds (samples OTN2 and MTN5) and soil (samples OTS3 and MTS6).

## Discussion

The profiling of bacterial communities indicated that the termite gut environment harbor bacterial communities that are unique and different to those of soil environment. There was a significant difference in the bacterial composition and community structure between the guts and savannah soils as indicated by the distribution of the major bacterial phyla (*Bacteroidetes*, *Actinobacteria*, *Proteobacteria*, *Spirochaetes*, *Firmicutes*, *Fibrobacters* and *Acidobacteria*) and genera (Fig. 1a; Additional file 2) across the samples. The gut samples of the fungus-feeders were dominated by members of *Bacteroidetes*, which were detected to a lesser extent in the mound and surrounding soil samples. Interestingly, the gut *Bacteroidetes* were mainly from members of the class *Bacteroidia* while those from soil samples belonged primarily to the *Flavobacteria*, *Cytophagia*, and *Sphingobacteria* classes. The genera *Dysgonomonas*, *Parabacteroides*, *Paludibacter*, *Tannerella*, *Alistipes* and *BCf9*-*17 termite group* were the major genera, but were rarely detected in the mounds and savannah soil (Additional file 2). These genera may represent the termite-specific bacterial lineages reported in other termites (Shinzato et al. 2007; Otani et al. 2014). The relatively low abundance of *Bacteroidetes* in the gut of the *Microcerotermes* sp. was evident. Members of *Bacteroidetes* are thought to be specialized in degradation of complex organic matter in the biosphere (Church 2008), implicating their roles in plant biomass degradation.

Moreover, the dominancy of *Spirochaetes* in the gut of *Microcerotermes* species was more pronounced than in the gut of fungus-feeders and soil samples. This demonstrates that members of *Spirochaetes* are host-associated and form an abundant group in the gut of wood-feeding termites (Köhler et al. 2012), where they are thought to be responsible for H_2_ production (Graber et al. 2004). Notably, *Spirochaetes* are rarely detected in fungus-feeding termites, ranging from almost absence in *Macrotermes* species (Otani et al. 2014), to approximate 10 % in *Odontotermes* species (Liu et al. 2013; Makonde et al. 2013a; Otani et al. 2014) and 22–36 % in *Microtermes* species (Makonde et al. 2013a; Otani et al. 2014). Again, *Spirochaetes* have been insignificantly detected within the mounds (Fall et al. 2007) and soils (Nacke et al. 2011).

The abundance of members of *Actinobacteria* within the mounds is noteworthy since the savannah soil was dominated by members of the group *Acidobacteria*. Importantly, are the genera *Arthrobacter, Nocardioides*, *Streptomyces* and *Solirubrobacter* that were not only relatively abundant in the mounds (Additional file 2), but are potential candidates for bioremediation (Shi et al. 2011). Besides, *Actinobacteria* have been demonstrated to produce antimicrobial compounds, which partly help prevent contamination in the farming of fungus gardens (Moriya et al. 2005), by inhibiting growth of some *Pseudoxylaria* and *Termitomyces* species (Visser et al. 2012).

The members of *Proteobacteria* were relative abundant in the mounds and savannah soil, however, they did not form the dominant group. This may be due to changes in soil properties, especially pH that has been shown to negatively influence the abundance of some *Proteobacteria* subdivisions (Nacke et al. 2011). Contrarily, members of the phylum *Proteobacteria* were represented by relative low abundances in the termite guts despite being known to have crucial role. For instance, members of *δ*-*Proteobacteria* such as *Desulfovibrio* spp. isolated from termite guts display high rates of H_2_-dependent oxygen reduction (Kuhnigk et al. 1996). The relative abundance of members belonging to the phylum *Firmicutes*, especially the class *Clostridia* was higher in the gut compared to the mounds and savannah soil, which contradicts the results of Fall et al. (2007) who reported relatively high abundance of *Firmicutes* in the mound belonging to soil-feeding termites. The relative high abundance of members of the phylum *Fibrobacteres* in *Microcerotermes* sp. may be associated with the degradation of plant-based cellulose (Qi et al. 2005), which is the main diet for these termites.

The bacterial composition between the mounds and savannah soil showed low variation, as demonstrated by weak grouping of samples (Fig. 3). Studies indicate that discrepancies in community structures between termite mounds and surrounding soil could be attributed by the trophic and mound construction behavior of the termites (Harry et al. 2001; Fall et al. 2007). The use of feces in building materials by termites may create an environment conducive to the development or the sustenance of particular microorganisms (Harry et al. 2001). Besides, the presence of clay may offer protection to some microorganisms (Harry et al. 2001) thereby increasing their survival. The tendency of increase in clay content in the mounds than the surrounding soil samples may demonstrate that soils in the mounds are enriched with clay particles due to the preferred selection of clay particles by termites (Manuwa 2009; Muwawa et al. 2014).

## Conclusions

The findings from this study have revealed diverse bacterial communities in the gut and surrounding soil environments, the majority of which are uncharacterized. We also note tendency of increase in clay particles in the mounds, which may be one of the factor influencing the prevalence of bacterial communities in the investigated environments. The bacterial community composition and structure in gut and soil environments were different but that of mound and surrounding soil were negligible. Although the methodology applied in this study cannot help infer physiological roles for the uncultured bacteria, the data obtained contribute to understanding the bacterial diversity and community structure in the gut and surrounding soil environments.

## Methods

### Research authorization

The Research Authorization was obtained from National Commission for Science, Technology and Innovation (NACOSTI) in Kenya. Kenya Wildlife Services (KWS) and the National Environmental Management Authority (NEMA) of Kenya approved the research and provided permits and other necessary documents for sample collection in Kenya.

### Site description and Sampling

The samples used in this study were collected from Thika district, Kenya (latitude 1°5′54.68″N, longitude 37°1′1.10″W) as described elsewhere (Makonde et al. 2013b). Termite mounds (C and D, approximately 2 km far apart were colonized by *Odontotermes* sp. [JQ247986] (OTG1) and *Macrotermes michaelseni* [JQ247993] (MTG4) together with *Microtermes* sp. [JQ247990] (MIG7), respectively) were excavated to a depth of 0.5–1.0 m. Next to mound D [about 2 m a way, was a colony of a wood feeding termite species (*Microcerotermes* sp. (MCG8)] that was also collected and analyzed. Termites (n = 200 workers and 50 soldiers) were sampled into sterile plastic boxes. Worker-caste termites were used in the experiments due to their foraging behaviour. The identity of the termites was confirmed by sequencing the mitochondrial cytochrome oxidase II gene in DNA extracted from the heads of soldiers (Makonde et al. 2013b) and comparing it to the sequences of previously identified specimens (Inward et al. 2007). In addition, soil samples (~40 g collected at ~5 cm depth) from the termite mounds C (OTN2) and D (MTN5) and their surrounding soils samples (OTS3 and MTS6, collected 3 m away from each mound, respectively), were included in the analyses.

### Physico-chemical analyses

Standard physical soil analyses, which involved texture and bulk density analyses of the soil samples, were performed according to Ackerman et al. (2007). Particle size distribution was determined by the hydrometer method for determining the silt and clay fraction as described by Manuwa (2009). Determination of pH and inorganic nitrogen of the samples were performed according to the methods described by Muwawa (2014). Carbon content was determined by the WalkleyBlack method (Walkley and Black 1934) while nitrate concentrations was determined by colorimetric method (Muwawa 2014).

### DNA extraction

The exterior surfaces of the termites were washed with 70 % ethanol and then rinsed with sterile distilled water. The guts were aseptically removed with forceps (Schmitt-Wagner et al. 2003). A total of 165 guts (approximately 1 g) of the *Odontotermes* sp. (OTG1) and *Macrotermes michaelseni* (MTG4) and 198 guts (approximately 1 g) of *Microtermes* sp. (MIG7) and 176 guts (approximately 1 g) of *Microcerotermes* sp. (MCG8) were put separately into sterile micro tubes containing 0.5 ml of TE buffer (10 mM Tris–HCl, 1 mM EDTA, pH 8.0). They were then homogenized using a sterile glass rod. The corresponding homogenates were then transferred into sterile tubes and used for total DNA extraction. The soil samples were homogenized separately and coarse stones and roots were removed. Subsequently, soil samples (~4 g) were used for total microbial DNA extraction. Total DNA extraction for all samples was performed using MoBio PowerMax Soil DNA isolation kit (MoBio Laboratories, Inc. CA, USA) according to the manufacturer’s protocol. DNA concentration was quantified by using a NanoDrop Spectrophotometer (NanoDrop Technologies, USA) as recommended by the manufacturer.

### Amplification of 16S rRNA genes and 454-pyrosequencing

The V3–V5 region of the bacterial DNA was PCR amplified using the universal 16S rRNA primers 357F (5′-TACGGRAGGCAGCAG-3′) (Wilson et al. 1990) and 926R (5′-CCGTCAATTCMTTTGAGT-3′) (Muyzer et al. 1996). The bacterial primers were modified for 454 pyrosequencing by attaching an Adaptor sequence, a key and a unique 12 Nucleotide MID for multiplexing purposes (Caporaso et al. 2010). Each PCR reaction (50 μL) contained forward and reverse primers (10 μM, each), dNTP’s (10 mM each), Phusion GC buffer (Finzymes), Phusion high fidelity polymerase (0.5 U μL^−1^) and 25 ng of template DNA. Cycling conditions were as described by Nacke et al. (2011); however for the bacterial DNA annealing was at 66 °C for 45 s. Amplification was confirmed by separating 2 µL of the PCR product on a 1 % TAE agarose gel (40 mM Tris base, 20 mM glacial acetic acid, 1 mM EDTA, 1.5 % (w/v) agarose run for 1 h at 100 V. Later three independent PCR products per sample were pooled in equal amounts, separated on a gel and extracted using the peqGOLD gel extraction kit (PeqLab Biotechnologie GmbH, Erlangen, Germany). Quantification of the PCR products was performed by using the Nanodrop (NanoDrop Technologies, USA) method and a Qubit fluorometer mbH, (Invitrogen GmbH Karlsruhe, Germany) as recommended by the manufacturer. Sequencing of the PCR amplicons was done at the Göttingen Genomics Laboratory using Roche GS-FLX 454 pyrosequencer (Roche, Mannheim, Germany) as recommended in the instructions of the manufacturer for amplicon sequencing.

### Pyrosequencing data analysis

Raw sequence reads were quality filtered according to the published recommendations (Huse et al. 2007) using the QIIME release 1.5.0 (Caporaso et al. 2010). The denoised sequences (≥300 bp) were then evaluated for potential chimeric sequences using UCHIME within the USEARCH package v.4.2.66 (Edgar 2010). A sequence identity cutoff of 97 % was used to pick OTUs from the quality filtered non-chimeric sequences. Representative OTUs were picked using the de novo OUT clustering (Rideout et al. 2014) with standard UCLUST method using the default settings as implemented in QIIME at 97 % similarity level. OTU alignment was done using the python implementation of the NAST algorithm, PyNAST (Caporaso et al. 2010). Taxonomy was assigned to the representative sequences from each cluster using BLASTn against the SILVA SSU Reference 119 database at default e-value threshold of 0.001 in QIIME (Quast et al. 2013) at dissimilarity levels of 3, 5 and 10 %. Rarefied datasets were generated with the multiple_rarefaction function in QIIME in order to remove sample heterogeneity before diversity assessment. Rarefaction curves and diversity indices were calculated and plotted for each sample using QIIME (Caporaso et al. 2010). To determine the amount of dissimilarity (distance) between any pair of bacterial communities, we used the UniFrac metric (Lozupone and Knight 2005; Lozupone et al. 2007) that incorporates the degree of divergence in the phylogenetic tree of OTUs into Principal coordinates analysis (PCoA). UniFrac distances are based on the fraction of branch length shared between two communities within a phylogenetic tree constructed from the 16S rRNA gene sequences from all communities being compared. A relatively small UniFrac distance implies that two communities are compositionally similar, harboring lineages sharing a common evolutionary history. In unweighted UniFrac, only the presence or absence of lineages is considered. We used the analysis of similarities (ANOSIM) (Clarke 1993; Fierer et al. 2010) through 1000 permutations to test for differences in community composition among the groups of samples. Additionally, the relative abundance of the genera were used in hierarchical clustering using the pearson correlation distance metric implemented in MultiExperimentViewer version 4.9.0 (MeV 4.9.0). The relative abundances (≥2.2 % at least in one sample) of the cultivated genera were also used for correlation analysis using Principal Components Analysis (PCA) as implemented in XLSTAT version 2015.4.01. All pyrosequencing-derived 16S rRNA gene sequences datasets were deposited in the GenBank under accession number SRP019764.

## Additional files

Additional file 1:
**a.** Physical and chemical characteristics of the analyzed soil samples. **b.** Rarefaction curves indicating the observed number of operational taxonomic units (OTUs). (I) Indicates observed number of OTUs at 5 % genetic distance. (II) Indicates observed number of OTUs at 10 % genetic distance. Key: The samples are marked by different colors. MCG8, *Microcerotermes* sp. gut homogenate; MIG7, *Microtermes* sp. gut homogenate; OTG1, *Odontotermes* sp. gut homogenate; MTG4, *M. michaelseni* gut homogenate; OTN2, Soil from mound C of *Odontotermes *sp.; MTN5, Soil from mound D of *M. michaelseni*; MTS6, Soil collected 3m away from mound D; OTS3, Soil collected 3m away from mound C. Additional file 2. Relative abundances of bacterial groups across the samples. Key: MCG8, *Microcerotermes* sp. gut homogenate; MIG7, *Microtermes* sp. gut homogenate; OTG1, *Odontotermes* sp. gut homogenate; MTG4, *M. michaelseni *gut homogenate; OTN2, Soil from mound C of *Odontotermes *sp.; MTN5, Soil from mound D of *M. michaelseni*; MTS6, Soil collected 3m away from mound D; OTS3, Soil collected 3m away from mound C.
