# Supplementary material for: 454 Pyrosequencing-based assessment of bacterial diversity and community structure in termite guts, mounds and surrounding soils
Source: Springerplus. 2015 Sep 2;4:471. doi: 10.1186/s40064-015-1262-6 (PMC4556716; doi:10.1186/s40064-015-1262-6)
Supplement: Additional file 1: — a. Physical and chemical characteristics of the analyzed soil samples. b. Rarefaction curves indicating the observed number of operational taxonomic units (OTUs). (I) Indicates observed number of OTUs at 5 % genetic distance. (II) Indicates observed number of OTUs at 10 % genetic distance. Key: The samples are marked by different colors. MCG8, Microcerotermes sp. gut homogenate; MIG7, Microtermes sp. gut homogenate; OTG1, Odontotermes sp. gut homogenate; MTG4, M. michaelseni gut homogenate; OTN2, Soil from mound C of Odontotermes sp.; MTN5, Soil from mound D of M. michaelseni; MTS6, Soil collected 3m away from mound D; OTS3, Soil collected 3m away from mound C. [file 40064_2015_1262_MOESM1_ESM.doc]

**Additional file 1a** Physical and chemical characteristics of the analyzed soil samples.

|  |  |  |  |  |  | **Texture (% dry soil)** | | |
| --- | --- | --- | --- | --- | --- | --- | --- | --- |
| **Sample ID** | **Sample description** | **pH** | **OC (mgg^-1^)** | **Total N (mgg^-1^)** | **C:N ration** | **Clay** | **Silt** | **Sand** |
| OTG1 | *Odontotermes* sp. gut homogenate | 7.2 | ND | ND | ND | ND | ND | ND |
| OTN2 | Soil from mound C of *Odontotermes* sp. | 6.4 | 2.05 ± 0.11 | 0.21 ± 0.01 | 9.76 | 30.00 | 3.40 | 66.60 |
| OTS3 | Soil collected 3 m away from mound C | 5.8 | 2.91 ± 0.08 | 0.27 ± 0.01 | 10.78 | 27.50 | 3.40 | 69.10 |
| MTG4 | *M. michaelseni* gut homogenate | 7.5 | ND | ND | ND | ND | ND | ND |
| MTN5 | Soil from mound D of *M.*  *michaelseni* | 6.6 | 1.88 ± 0.11 | 0.17 ± 0.01 | 11.06 | 20.00 | 5.90 | 74.10 |
| MTS6 | Soil collected 3 m away from mound D | 5.6 | 2.99 ± 0.08 | 0.29 ± 0.01 | 10.31 | 2.50 | 3.40 | 94.10 |
| MIG7 | *Microtermes* sp. gut homogenate | 7.6 | ND | ND | ND | ND | ND | ND |
| MCG8 | *Microcerotermes* sp. gut homogenate | 7.7 | ND | ND | ND | ND | ND | ND |

**Additional file 1b** Rarefaction curves indicating the observed number of operational taxonomic units (OTUs). (I) Indicates observed number of OTUs at 5% genetic distance. (II) Indicates observed number of OTUs at 10% genetic distance.

**(I)**


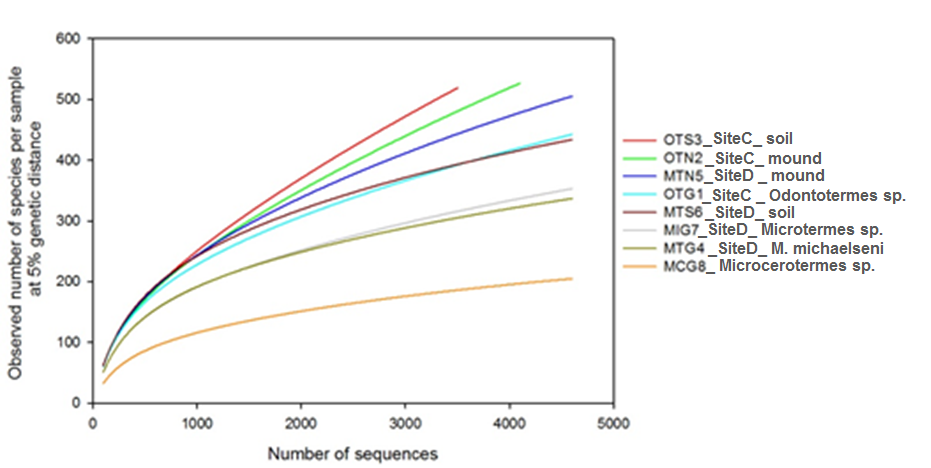


**(II)**


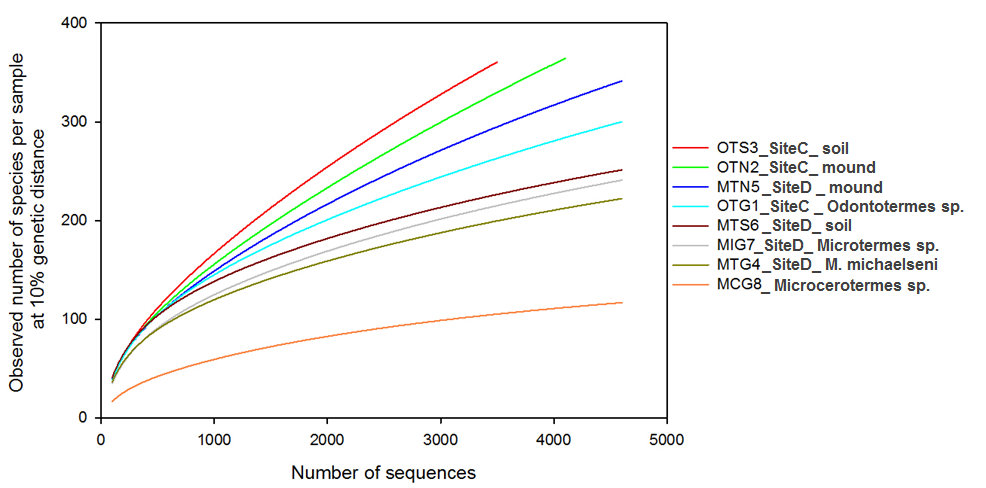


Key: The samples are marked by different colors. Key: MCG8, *Microcerotermes* sp. gut homogenate; MIG7, *Microtermes* sp. gut homogenate; OTG1, *Odontotermes* sp. gut homogenate; MTG4, *M. michaelseni* gut homogenate; OTN2, Soil from mound C of *Odontotermes* sp.; MTN5, Soil from mound D of *M.*  *michaelseni* ; MTS6, Soil collected 3 m away from mound D; OTS3, Soil collected 3 m away from mound C.
